# Utilizing Exosomal-EPHs/Ephrins as Biomarkers and as a Potential Platform for Targeted Delivery of Therapeutic Exosomes

**DOI:** 10.3390/ijms23073551

**Published:** 2022-03-24

**Authors:** Dimitrios Goutas, Alexandros Pergaris, Nikolaos Goutas, Stamatios Theocharis

**Affiliations:** 1 First Department of Pathology, Medical School, National and Kapodistrian University of Athens, 75, Mikras Asias Street, Bld 10, Goudi, 11527 Athens, Greece; alexperg@yahoo.com (A.P.); stamtheo@med.uoa.gr (S.T.); 2 Department of Forensic Medicine and Toxicology, Pathology, Medical School, National and Kapodistrian University of Athens, 75, Mikras Asias Street, Goudi, 11527 Athens, Greece; ngoutas@med.uoa.gr

**Keywords:** EPHs, ephrins, exosomes, cancer, therapy, biomarkers, prognosis

## Abstract

Exosomes are cell-secreted nanoparticles containing various molecules including small vesicles, microRNAs (miRNAs), messenger RNAs or bioactive proteins which are thought to be of paramount importance for intercellular communication. The unique effects of exosomes in terms of cell penetration capacity, decreased immunogenicity and inherent stability, along with their key role in mediating information exchange among tumor cells and their surrounding tumor microenvironment (TME), render them a promising platform for drug targeted delivery. Compared to synthetic drugs, exosomes boast a plethora of advantages, including higher biocompatibility, lower toxicity and increased ability of tissue infiltration. Nevertheless, the use of artificial exosomes can be limited in practice, partly due to their poor targeting ability and partly due to their limited efficacy. Therefore, efforts have been made to engineer stem cell-derived exosomes in order to increase selectiveness and effectivity, which can then become loaded with various active substances depending on the therapeutic approach followed. Erythropoietin-producing human hepatocellular receptors (EPHs), along with their ligands, the EPH family receptor interacting proteins (ephrins), have been extensively investigated for their key roles in both physiology and cancer pathogenesis. EPHs/ephrins exhibit both tumorigenic and tumor suppressing properties, with their targeting representing a promising, novel therapeutic approach in cancer patients’ management. In our review, the use of ephrin-loaded exosomes as a potential therapeutic targeted delivery system in cancer will be discussed.

## 1. Introduction

Erythropoietin-producing human hepatocellular receptors (EPHs) compose the largest known subfamily of receptor tyrosine kinases. They participate, along with their ligands, with the EPH family receptor interacting proteins (ephrins) in a wide range of processes in human physiology [1]. EPHs are membrane-bound proteins consisting of an extracellular ephrin-binding domain, a transmembrane region and a cytoplasmic component. Ephrins also comprise membrane-bound proteins, therefore, cell-to-cell interaction is required for an EPH to interact with its ligand. Following EPH–ephrin interaction, a response is triggered in not only the cytoplasm of the EPH-expressing cell (a process called forward signaling) but also in the cytoplasm of the ephrin-bearing one (termed reverse signaling). The message is further transmitted through complex molecular cascades implicated in both processes [2].

**Figure 1:**
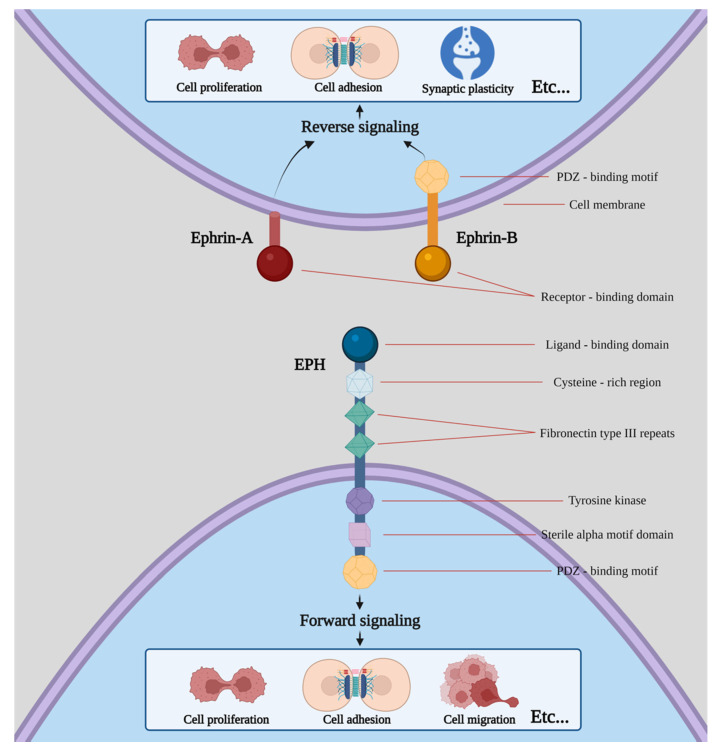
Structure of EPHs/ephrins and schematic presentation of forward and reverse signaling. Etc. means that both procedures can induce a plethora of effects apart from the ones shown in the picture, including cell segregation, border sharpening, cell repulsion and neurite outgrowth as well as cell survival and maturation.

Nine EPHA receptors (EPHA1 to EPHA8 and EPHA10) that bind 5 ephrin-A ligands (ephrin-A1 to ephrin-A5), along with five EPHB receptors (EPHB1 to EPHB4 and EPHB6) that interact with three ephrin-B ligands (ephrin-B1 to ephrin-B3) are expressed in humans [3,4,5,6]. While a higher affinity between receptors and ligands of the same subgroup is observed, crosstalk between different subgroups has also been described (Figure 1).

The EPH/ephrin system participates in, among other processes, cell migration, axon guidance and synapse formation during embryonic development as well as procedures such as like-cell adhesion, motility, cell–matrix interactions, lymphangiogenesis and angiogenesis [7]. As most of the aforementioned procedures comprise key steps of carcinogenesis, EPHs/ephrins have been extensively investigated so that their role in neoplasia can be clearly elucidated [8].

Meanwhile, exosomes represent a type of extracellular vesicle (EV) containing biomolecules such as proteins, mRNAs, microRNAs (miRNAs), polysaccharides and lipids of the cells that secrete them. While the physiological role remains largely unknown, it has been speculated that they have a role in removing excess and/or unnecessary constituents from cells to maintain cellular homeostasis. Nevertheless, since they possess the ability to mediate intercellular communications, they have been utilized as nanocarriers for drug delivery [9]. Exosomes isolated from a patient’s own cells have higher biocompatibility and lower toxicity compared with synthetic drugs and they are capable of penetrating into tissues, diffusing into the blood and even crossing the blood–brain barrier [10]. Furthermore, exosome-mediated delivery can bypass the P-glycoprotein drug efflux system and, as a result, reduce drug resistance [11].

Exosomes are usually smaller than other extracellular vesicles and therefore require special handling in their separation and analysis [12]. Nevertheless, despite the variety of techniques and approaches that have been used for the purification, separation and analysis of exosomes, so far, no methodology providing enough insight regarding selectivity, purification yield and reproducibility exists. In fact, due to their biochemical properties, a combination of techniques usually needs to be tailored to obtain the desired purification outcomes. Since exosomes must be isolated from varied biological samples while preserving their physiochemical properties and biological function, and as they exhibit significant heterogeneity in size, cargo and surface markers, their isolation needs to be specific, efficient and have long-term perspective of clinical applications. Taking these into consideration, current methods of isolation include ultracentrifugation, filtration, precipitation, chromatography, microfluidics and immunoaffinity capture [13]; among them, ultracentrifugation represents the gold standard for exosome isolation [14].

Considering the advantages that exosomes confer as a therapeutic platform for drug targeted delivery along with the constantly rising associations of EPH/ephrins with a variety of diseases ranging from pathological processes to cancer, this makes them an attracting therapeutic approach. Furthermore, various studies have demonstrated the important role of circulating exosomal EPHs/ephrins in different disease states, solidifying their part as a biomarker to be further investigated. That said, the use of EPHs/ephrins, either in the form of circulating them, bound to exosomes, or through directly loading them to exosomes, can become utilized as a therapeutic strategy and/or as a prognostic biomarker.

## 2. Exosomes and Tumor Microenvironment

The tumor microenvironment (TME) is comprised of diverse cell types in a variety of functional niches, constituting a complex “ecosystem” and a vital contributor in cancer initiation and progression that ultimately modulates a plethora of cell-to-cell interactions [15,16]. The aforementioned interactions orchestrate reprogramming into cancer-permissive environments and can have significant impacts on cancer development. Furthermore, intercellular communication in the TME, through a variety of signaling networks, allows for information exchange to occur among cells ranging from juxtacrine interactions to secreted factors, such as exosomes [17]. Exosomes and other EVs underline the complexity of dynamic cell-to-cell interactions that form TME. In a similar fashion, Zhao et al. [18] studied the exosomes derived from cancer-associated fibroblasts (CAFs). They observed that CAF-derived exosomes could inhibit mitochondrial oxidative phosphorylation, thereby increasing glycolysis and glutamine-dependent reductive phosphorylation in tumor cells. They further proved, through intra-exosomal metabolomics, that CAF-derived exosomes consist of intact metabolites, including amino acids, lipids and TCA-cycle intermediates, which are ultimately utilized by cancer cells for promoting tumor growth under nutrient deprivation or nutrient stressed conditions [18]. A number of studies have established the hypoxic tumor microenvironment as a common feature of solid tumors, linked with tumor aggressiveness and poor patient prognosis [19,20]. Exosomes mediate and assist in the continuous crosstalk among tumor and stromal cells and are believed to regulate hypoxia adaptation and to rebuild the microenvironment in return [21]. Nevertheless, what is worth mentioning is the potential that exosomes carry as indicators of tumor burden and prognosis and as a potential therapeutic treatment, as they regulate a variety of aspects of heterotypic cell-to-cell interaction within TME.

## 3. The Role of Exosomes

### 3.1. Exosomal Engineering and Loading

**Figure 2:**
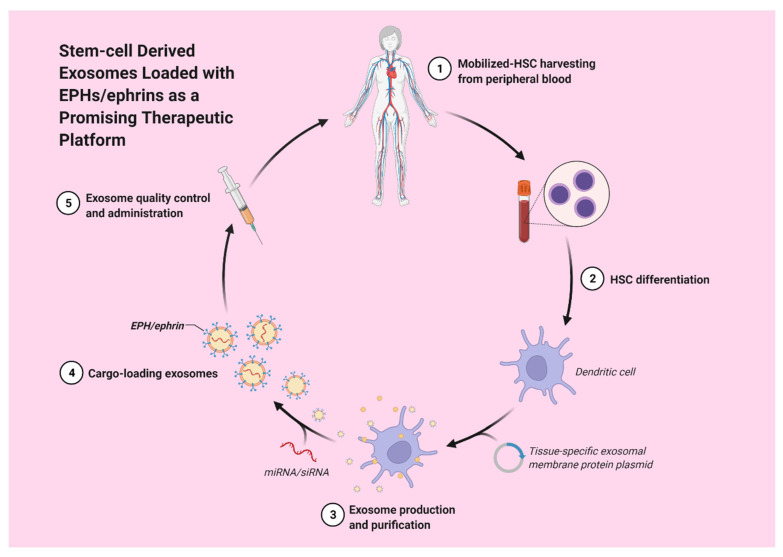
Stem-cell derived exosomes loaded with EPHs/ephrins could potentially act as a promising therapeutic strategy.

Hematopoietic stem cell-derived exosomes have been found to express mRNAs of several pro-angiopoietic and anti-apoptotic factors such as insulin growth factor 1, vascular endothelial growth factor (VEGF), interleukin 8 and basic fibroblast growth factor [22]. These mRNAs have the ability to enhance endothelial cells’ proliferation and survival, exert anti-apoptotic effects and, thereby, stimulate tube formation [22]. Moreover, exosomes derived from a patient’s differentiated hematopoietic stem cells can be used for tissue-targeted cargo delivery through the expression of tissue-specific peptides. By loading miRNA or siRNA of the targeted gene, exosomes can selectively regulate gene expression (Figure 2) [23].

Mesenchymal stem cell (MSC)-derived exosomes have been widely investigated in a variety of disease states and have been shown to be capable of immunoregulation, regenerating tissue and promoting angiogenesis [24]. Employing the advantages of MSC-exosomes, they can be loaded with miRs and targeted to the desired site. Mardpour et al. [24] utilized hydrogel-mediated MSC-derived exosomes loaded in patients with chronic liver failure, managing to augment liver regeneration. An in vivo study, conducted by O’Brien et al. [25], used MSC-derived exosomes loaded with a potential tumor suppressor, miR-379, for breast cancer therapy. It was found that miR-379 indeed acted as a potent tumor suppressor in breast cancer, partly attributed to its interaction with COX-2.

In order to confer cell type targeting sensitivity, modification strategies of exosomes include genetic engineering and chemical modification [26,27,28]. In genetic engineering, the gene sequence of a guiding protein or polypeptide is fused with the preferred exosomal membrane protein. Via this approach, surface display of peptides and proteins is achieved, with, however, a limitation on targeting motifs that are genetically encodable. On the other hand, through chemical modification, a wide range of ligands, natural and synthetic, can be displayed via lipid assembly or conjugation reactions. The latter have the advantage to stably adjust exosomal surface proteins, but the complex exosomal surface may compromise the reaction efficiency [28]. Covalent modification may also risk the function of the exosome. Moreover, lipids and amphipathic molecules can become enclosed in the lipid bilayer of exosomes and allow their hydrophilic part to be displayed on the exterior, ultimately causing an increase in the toxicity of exosomes [28].

Proteomics data (present in databases such as ExoCarta and Vesiclepedia), studied to identify the components of exosomes, have shown that all EPH receptors and ephrinB proteins have been detected in exosomes purified from body fluids and normal cells as well as from a broad range of cancer cell types [29,30,31]. Nevertheless, the possibility of exploiting the EPH system for targeted delivery of therapeutic exosomes either through purification or genetic engineering has not yet been studied.

### 3.2. Exosomal EPHs Targeted Delivery

Targeted delivery systems represent the backbone of personalized medicine to surpass the toxic effects and the seemingly insurmountable obstacle of off-target implications. Emerging data have shed light to the paramount role of exosomes in disease and on their ability to carry proteins to distant tissue locations where they exert their properties [29,30,31,32,33,34,35,36,37,38,39,40,41,42,43,44,45,46]. Up until now, it was believed that for an EPH to interact with its ligand, a direct cell-to-cell interaction was required. However, Gong et al. [30] demonstrated in their studies that EPH receptors and ephrins were able to have a long-range intercellular communication and, paradoxically, still involve direct contact between two cell membranes [30]. This is possible through the integration of a member of the EPH/ephrin system in the membrane of the exosome. Following the release of the exosome, exosomal membrane-bound EPHs/ephrins can interact with their high affinity counterparts present on cell membranes of cells located at distant sites, a process that results in EPH–ephrin interaction without direct cell-to-cell membrane contact. This form of communication exploits the use of exosomes that are released by cells and are capable of travelling to distant sites via interstitial and other body fluids [8,9]. Therefore, taking into consideration the fact that EPH–ephrin signaling does not require direct cell contact [30], this makes the utilization of exosomal EPHs and ephrins paramount not just as a biomarker of disease progression, prognosis and carcinogenesis but also as a therapeutic approach for a wide variety of diseases.

### 3.3. The Role of Exosomal EPHs in Cancer

Many types of tumor cells carry and secrete exosomes, containing miRNAs and functional proteins, to stroma cells, ultimately leading to tumor angiogenesis enhancement along with an augmented rate of extracellular matrix degradation and remodeling and accelerated tumor stromal invasion [42]. These facts highlight the role of exosomes in expediting tumor development (Table 1).

### 3.4. Angiogenesis

Several studies have revealed an association between members of the EPH/ephrin system, as a component of cancer-derived exosomes and angiogenesis [42,43,44]. Sato et al. [44] studied the role of small extracellular vesicles (SEVs), in the size range of exosomes, derived from head and neck squamous cell carcinoma (HNSCC) in promoting tumor angiogenesis. Western blot analyses of exosomes and patient data analyses revealed that EPHB2 was overexpressed in HNSCC patients and was associated with poor patient prognosis and tumor angiogenesis. Moreover, functional experiments demonstrated that EPHB2 expression in exosomes modulated angiogenesis. Meanwhile, exosomal EPHB2 stimulated ephrin-B reverse signaling by inducing STAT3 phosphorylation [44]. Additionally, EPHA2 has been implicated in enhancing angiogenesis via proteomic analysis of lung tumor cell-derived exosomes, while an inhibition assay revealed that EPHA2 constitutes a major MAPK activator on exosomes [42]. More specifically, it was demonstrated that the direct communication among membrane protein (EPHA2) on exosomes and recipient cells resulted in stimulation of tumor endothelial cells [42]. These results highlight the fact that EPHA2 participates in angiogenesis as a ligand of the ephrin signaling pathway [42]. Furthermore, Wang H. et al. [43] attempted to provide an association on how oral squamous cell carcinoma (OSCC) affects the angiogenesis of human umbilical vein endothelial cells (HUVECs) via miR-210-3p expression and divulge the relationship between miR-210-3p, its target protein and the mechanism of angiogenesis regulation [43]. They observed that EPHA3 was the target gene of miR-210-3p and that its protein levels could influence the migration and proliferation of HUVECs. In addition, by measuring the levels of phosphorylated AKT in HUVECs, they demonstrated that when EPHA3 was downregulated, the AKT levels were elevated, while when EPHA3 was upregulated, the PI3/AKT pathway was suppressed. When concluding their results, Wang H. et al. proved that the exosomes secreted by OSCC cells could upregulate the expression of miR-210-3p while reducing EPHA3 expression in HUVECs and promoting tube formation via the activation of PI3/AKT signaling pathway [43].

### 3.5. Chemoresistance Transmission

Fan et al. [40] attempted to study the mechanisms of tumor chemoresistance in pancreatic cancer (PC) via utilizing in vitro cell cultures to characterize the ability of PC derived exosomes to increase resistance to gemcitabine [40]. They observed that three PC cell lines, PANC-1, BxPC-3 and MIA PaCa-2 (Panc-1-highly metastatic cell line and MIA PaCa-2 and BxPC-3-low metastatic cell lines), displayed distinctive resistance to gemcitabine, with PANC-1 cells showcasing significantly greater chemoresistance to gemcitabine. After isolating exosomes from PANC-1 and MIA PaCa-2 and BxPC-3 cell lines, in order to establish whether exosomes derived from PANC-1 (gemcitabine resistant) cells can convey resistance to BxPC-3 and MIA PaCa-2 (gemcitabine sensitive) cells, they incubated the above, gemcitabine sensitive cell lines with PANC-1 exosomes for 24 h. As a result, PANC-1 exosome treatment notably augmented gemcitabine resistance of the BxPC-3 and MIA PaCa-2 cell lines in accordance with the exosome dose administered [40]. Their findings reflect the theory that PC tumor exosomes have the ability to convey chemoresistance to PC sensitive cells within the same tumor or at other distant anatomic sites [40]. Furthermore, they determined that it is exosomal EPHA2 that mediates transmission of gemcitabine resistance to gemcitabine sensitive cells [40], highlighting its role in drug resistance and as a potential biomarker in PC.

Furthermore, Ji H. et al. [38] analyzed the secretome protein profiles released in vitro from isogenic human colorectal cancer (CRC) cells; the analysis resulted in observing the selective enrichment of the metastatic CRC cell exosomes with key metastatic factors (MET, S100A8, S100A9, TNC) and signal transduction molecules (EFNB2, EGFR, JAG1, SRC, TNIK) relative to primary CRC cell exosomes [38]. As a result, numerous proteins that have selectively been enriched in metastatic CRC cell-derived exosomes can function both as metastatic factors and as important signaling pathways, ultimately enhancing our knowledge on the crosstalk between tumor and stromal cells in the TME [38].

Another study, performed by Jung et al. [36] in an effort to elucidate the role of exosomes in neoplastic cells, demonstrated that exosomes from hypoxic tumor cells can transfer miR-210 to normotoxic tumor or endothelial cells and that exosomal miR-210 can inhibit its target genes and promote angiogenesis in recipient cells [36]. Furthermore, their data demonstrated that the exosomes from the hypoxic tumor-bearing mice had elevated miR-210 levels when compared to normal mouse serum, indicating that miR-210 from circulating exosomes can be used as a potential biomarker for hypoxic tumors and could affect nearby cells to produce a more favorable environment for tumor survival and that exosomal miR-210 from hypoxic tumor cells can be transferred to various types of recipient cells, such as immune cells, epithelial cells and mesenchymal stem cells [36]. EPHA3 contains an established role in vascular endothelial growth factor (VEGF) signaling and angiogenesis and represents a miR-210 target gene [47,48]. Results showed that EPHA3 levels were decreased by treatment with exosomes containing miR-210 while VEGF levels were increased. As a result, and since EPH3 has an important role in vascular remodeling development, EPHA3 downregulation, through exosomal miR-210, could be correlated with angiogenic responses yielding in the genesis of new tubular structures and capillaries [36].

### 3.6. Senescent Cells Release Exosomes Contributing to Cancer Cell Proliferation

Although cell senescence prevents the proliferation of cells at risk for neoplastic transformation, the altered secretome that senescent cells develop can promote and contribute to cancer cell proliferation. Takasugi et al. [46] demonstrated that sEVs carrying EPHA2 bind to ephrin-A1 that is overexpressed in a variety of cancer cells [8] and ultimately augments their proliferation via the EPHA2/ephrinA1 reverse signaling [46]. They further investigated the mechanism of enrichment of EPHA2 in sEVs, secreted from senescent cells, considering some post-translational modification of EPHA2 to be involved. They examined and concluded that tyrosine kinase phosphorylation of EPHA2 in senescent cells, resulting from oxidative inactivation of PTP1B phosphatase, is involved in its sEV sorting [46]. As a result, that reactive oxygen species (ROS)-regulated cargo sorting into sEVs could prove to be critical for the possibly detrimental growth-promoting effect of the senescent cells’ secretome [46].

## 4. The Role of Exosomal EphA2 in Tumor Progression, Metastasis and Drug Resistance

A study performed by Wei et al. [45] utilized two PC cell lines in an effort to identify new diagnostic markers and understand the mechanism of PC progression [45]. After isolating, purifying and characterizing, in terms of morphology and exosomal markers expression levels, the exosomes derived from PC cells, Wei et al. analyzed the exosomal proteins. They observed that EPHA2 was overexpressed in Panc-1 cell lines compared with BxPC3 cells, indicating that the metastatic effects of Panc-1 exosomes are likely mediated by EPHA2 [45]. Furthermore, they obtained serum samples from 40 patients with PC and they quantified the exosomal EPHA2 levels. They found that exosomal EPHA2 levels were higher in patients with PC compared to healthy controls [45]. Taking the above into consideration, exosomal EPHA2 could be a potential oncogene in PC and be utilized as a potential tumor marker for PC diagnosis. Exosomal EPHA2 has also been linked to promoting breast cancer metastasis [32]. Gao et al. [32] demonstrated that drug resistant cell-derived exosomes promoted the invasion of sensitive breast cancer cells. Through quantitative proteomic analysis, they proved that EPHA2 was rich in exosomes from drug resistant cells and that exosomal EPHA2 conferred the invasive/metastatic phenotype transfer from drug resistant cells to sensitive cells [32]. Moreover, they demonstrated that exosomal EPHA2 could activate the ERK1/2 signaling through the ligand ephrin A1-dependent reverse pathway instead of the forward pathway, advocating, as a result, breast cancer progression [32]. Their results not only highlight the key role of exosomal EPHA2 in transmitting an aggressive phenotype among cancer cells, but also suggest that they do not rely, entirely, on direct cell-to-cell contact [32]. Furthermore, their findings propose that increased EPHA2 in drug-resistant cell-derived exosomes could present a principal mechanism of chemotherapy/drug resistance-induced breast cancer progression.

## 5. Tumor Innervation

Innervated tumors display a more aggressive phenotype, with increased metastasis and decreased survival of patients, as compared to the less innervated tumors [49,50,51]. In prostate cancer, engagement of nerve fibers to cancer tissue is directly linked to higher tumor proliferative indices and a higher risk of recurrence and metastasis [50]. Nevertheless, a mechanistic understanding of how tumors obtain their neural elements remains unclear. Although extracellular release of neurotrophic factors by cancer cells could also contribute to cancer progression [52,53], tumors also secrete additional components that may directly promote axonogenesis. Madeo et al. [33] utilized PC12 cells, a pheochromocytoma cell line, as an in vitro neuronal model, human tumor samples and murine in vivo models to test the hypothesis that, in some tumors, nerves are acquired by a tumor-induced process. They demonstrated that cancer exosomes released from patient tumors could stimulate PC12 cells and promote neurite outgrowth [33]. Furthermore, by using a cancer mouse model, they showed that tumors compromised in exosomes’ release are less innervated than controls and that in vivo pharmacological inhibition of exosome release attenuates tumor innervation in a similar fashion [33]. Many molecules contained as cargo in exosomes are potential candidates for exosome-induced axonogenesis. In their study, Madeo et al. [33] showed that tumor released exosomes with high ephrin–B1 content potentiated neurite outgrowth of PC12 cells, while inhibition of ephrin–B1 expression or function attenuated it. Taking these into consideration, their data suggest that cancer exosomes contribute to axonogenesis and that exosomal ephrin–B1 enhances this activity.

In another study, performed by Gong et al. [30], the interactome of clustered EPHB2 and identified members of endosomal sorting complex required for transport (ESCRT) as EPHB2 interactors was analyzed. They observed that exosomes from glioblastoma U-251MG cells and primary neurons contained endogenous EPHs and ephrins [30]. Meanwhile, exosomal EPHB2 was taken up by ephrinB1 cells, inducing ephrinB1 tyrosine phosphorylation and triggering neuronal growth cone collapse [30]. Their results suggest a mechanism of ephrin-EPH signaling, independent of direct cell contact and proteolytic cleavage, where EPHs and ephrins can signal at a distance through exosomes, in addition to the bidirectional signaling that depends on cell-to-cell contact, and, also, demonstrates the interaction of exosomal EPHB2 in neural development and synapse physiology.

## 6. Conclusions

Exosomes have widely been recognized as a potential and promising biomarker tool in cancer prognosis and diagnosis, owing to the large amount of biological information they carry. More specifically, molecular profiling of cancer exosomes, especially surface proteins and miRNAs, has been used in cancer diagnosis and treatment response monitoring [54,55]. Liang et al. [34] utilized a rapid, ultrasensitive and inexpensive nanoplasmon-enhanced scattering (nPES) assay that could directly quantify tumor derived exosomes from plasma through the use of antibody-conjugated nanospheres and nanorods [34]. They identified and demonstrated that exosomal EPHA2 could be utilized as a biomarker in PC for distinguishing PC from pancreatitis patients and healthy subjects [34]. Furthermore, they observed that exosomal EPHA2 could be informative in predicting tumor progression and in detecting early responses to neoadjuvant chemotherapy [34]. Since exosomal EPHs have been gaining attention regarding their interaction in cancer initiation and progression and regarding their use as potential biomarkers, the enhancement and enrichment of the techniques used for exosome separation and analysis of the biomarkers that they carry could bring more information about their function in different cancer types. Furthermore, as exosomes have the capability of transporting therapeutic genes, proteins and small molecules, they constitute an important vector for targeted delivery of ephrin molecules, enabling the therapy of various diseases with high efficiency and reduced toxicity. Thus, taking advantage of the EPH system for delivering therapeutic exosomes to cancer cells or other diseased cells overexpressing EPHs/ephrins could become a promising therapeutic platform for various diseases. However, the source of exosomes needs to be carefully considered, especially in the case of therapeutic application of drug or gene encapsulating exosomes, as different exosomal origins convey different features and compositions. Moreover, when investigating the side effects and treatment efficacy of exosomes, the possible risk of tumor growth induction by tumor cell-derived exosomes must be considered. Further research on the pharmacokinetic profile and biodistribution of exosomes needs to be carried out in order to proceed towards therapeutic utility of the engineered exosomes.

## Figures and Tables

**Table 1:** Delineating the mechanism of action and the effect of exosomal EPHs in various disease settings.

EPH/Ephrin	Tissue/Tumor Type	Methods	Mechanism	Effect	Ref.
EPHB2	Head and neck squamous cell carcinoma tissues	Western blot	Exosomal EPHB2 stimulated ephrin-B reverse signaling by inducing STAT3 phosphorylation	Angiogenesis (+overexpression of EPHB2 linked to poor patient prognosis)	[44]
EPHA2	lung tumors	proteomic analysis of lung tumor cell-derived exosomes	EPHA2 activates MAPK on exosomes	Angiogenesis	[42]
EPHA3	oral squamous cell carcinoma (OSCC)	measuring the levels of phosphorylated AKT in human umbilical vein endothelial cells (HUVECs)	(1) EPHA3 was the target gene of miR-210-3p and that its protein levels could influence the migration and proliferation of HUVECs (2) when EphA3 was downregulated, the AKT levels were elevated (3) when EphA3 was upregulated, the PI3/AKT pathway was suppressed (4) the exosomes secreted by OSCC cells could upregulate the expression of miR-210-3p while reducing EphA3 expression in HUVECs and promoting tube formation via the activation of PI3/AKT signaling pathway	Angiogenesis of HUVECs	[43]
EPHA2	pancreatic cancer with cell cultures of PANC-1, BxPC-3, MIA PaCa-2 cell lines	they incubated the above, gemcitabine sensitive cell lines, with PANC-1 exosomes for 24 h	N/D	tumor chemoresistance, three pancreatic cancer cell lines (PANC-1, BxPC-3, MIA PaCa-2) displayed distinctive resistance to gemcitabine, with PANC-1 cells showcasing significantly greater chemoresistance to gemcitabine	[40]
EFNB2	colorectal cancer cells	secretome protein profiles analysis	selective enrichment of the metastatic CRC cell exosomes with key metastatic factors (MET, S100A8, S100A9, TNC) and signal transduction molecules (EFNB2, EGFR, JAG1, SRC, TNIK) relative to primary CRC cell exosomes	tumor chemoresistance	[38]
EPHA3	N/D	N/D	EPHA3 contains an established role in vascular endothelial growth factor (VEGF) signaling and angiogenesis and represents a miR-210 target gene EPHA3 levels were decreased by treatment with exosomes containing miR-210 while VEGF levels were increased	angiogenesis	[47,48]
EPHA2	sEVs secreted from senescent cells	N/D	tyrosine kinase phosphorylation of EPHA2 in senescent cells, resulting from oxidative inactivation of PTP1B phosphatase is involved in its sEV sorting and augments their proliferation via the EPH2/ephrin-A1 reverse signaling	proliferation	[46]
EPHA2	two pancreatic cancer (PC) cell lines (Panc-1-highly metastatic cell line and BxPC-3-low metastatic cell line	N/D	EPHA2 was overexpressed in Panc-1 cell lines compared with BxPC3 cells	EPHA2 promotes metastasis	[45]
	serum samples from 40 patients with PC	N/D	EPHA2 levels were higher in patients with PC compared to healthy controls		
EPHA2	breast cancer cells	N/D	exosomes from drug resistant cells were rich in EPHA2 exosomal EPHA2 conferred the invasive/metastatic phenotype transfer from drug resistant cells to sensitive cells EPHA2 activates the ERK1/2 signaling through the ligand ephrin A1-dependent reverse pathway	drug resistance, invasion, metastasis	[32]
ephrin-B1	PC12 cells	N/D	exosomes with high EphrinB1 content potentiated neurite outgrowth of PC12 cells, while inhibition of EphrinB1 expression or function attenuated it	axonogenesis	[33]
EPHB2	glioblastoma U-251MG cells and primary neurons	N/D	EPHB2 interacts with identified members of endosomal sorting complex required for transport (ESCRT), EPHB2 was taken up by ephrin-B1 cells, inducing ephrin-B1 tyrosine phosphorylation and triggering neuronal growth cone collapse	axonogenesis	[30]

N/D: not determined.
